# Control of Newly-Designed Wearable Robotic Hand Exoskeleton Based on Surface Electromyographic Signals

**DOI:** 10.3389/fnbot.2021.711047

**Published:** 2021-09-15

**Authors:** Ke Li, Zhengzhen Li, Haibin Zeng, Na Wei

**Affiliations:** ^1^Laboratory of Rehabilitation Engineering, Research Center of Intelligent Medical Engineering, School of Control Science and Engineering, Shandong University, Jinan, China; ^2^Department of Radiotherapy, Suzhou Dushu Lake Hospital, Suzhou, China; ^3^Department of Geriatrics, Qilu Hospital, Shandong University, Jinan, China

**Keywords:** exoskeleton, surface electromyography, gesture recognition, wearable robots, hand rehabilitation

## Abstract

The human hand plays a role in a variety of daily activities. This intricate instrument is vulnerable to trauma or neuromuscular disorders. Wearable robotic exoskeletons are an advanced technology with the potential to remarkably promote the recovery of hand function. However, the still face persistent challenges in mechanical and functional integration, with real-time control of the multiactuators in accordance with the motion intentions of the user being a particular sticking point. In this study, we demonstrated a newly-designed wearable robotic hand exoskeleton with multijoints, more degrees of freedom (DOFs), and a larger range of motion (ROM). The exoskeleton hand comprises six linear actuators (two for the thumb and the other four for the fingers) and can realize both independent movements of each digit and coordinative movement involving multiple fingers for grasp and pinch. The kinematic parameters of the hand exoskeleton were analyzed by a motion capture system. The exoskeleton showed higher ROM of the proximal interphalangeal and distal interphalangeal joints compared with the other exoskeletons. Five classifiers including support vector machine (SVM), K-near neighbor (KNN), decision tree (DT), multilayer perceptron (MLP), and multichannel convolutional neural networks (multichannel CNN) were compared for the offline classification. The SVM and KNN had a higher accuracy than the others, reaching up to 99%. For the online classification, three out of the five subjects showed an accuracy of about 80%, and one subject showed an accuracy over 90%. These results suggest that the new wearable exoskeleton could facilitate hand rehabilitation for a larger ROM and higher dexterity and could be controlled according to the motion intention of the subjects.

## Introduction

The human hand plays a role in a variety of daily tasks. This delicate instrument is vulnerable to trauma or neurological or musculoskeletal disorders. Stroke, for example, could heavily affect hand function (Hu et al., 2018; Burns et al., 2019; Chowdhury et al., 2019). Over 85% of the post-stroke individuals reported that they could not control their hand freely for dexterous manipulation over 8 months after the onset of stroke. Hand rehabilitation, typically by intensive motor training for restoring hand function, would be one of the most urgent demands in post-stroke survivors, particularly for those who desire to maintain a high quality of life. Effective rehabilitation requires repetitive passive and active training, which could not be fully conducted face-to-face or hand-by-hand by therapists. A robotic hand exoskeleton can provide high training intensity, stable working performance, and adaptive movement assistance for functional training; thus it has been proven to be an effective technology for hand rehabilitation (Leonardis et al., 2015; Li et al., 2019). However, due to the compact dimensions, structural complexity, and high flexibility, developing a satisfactory hand exoskeleton and to freely control it in real-life scenarios is still a challenging task (Palm and Iliev, 2007).

From structure design, the hand exoskeleton could be divided into pneumatic, glove-based, and linkage-based exoskeletons. The pneumatic exoskeleton is easy to control, but is difficult to perform flexible finger movement (Gerez et al., 2020; Takahashi et al., 2020). Glove-based exoskeletons are usually more supple and comfortable to wear; but due to the coverage of the glove, it blocks the direct contact of the object and, thus, disturbs tactile feedback (Sarac et al., 2019). The linkage-based exoskeletons use mechanical links to connect the finger components, either by fingertip contact or by full contact with the hand. With fingertip contact, the advantage is that the position of the fingertip can be precisely controlled and, thus, suitable for all hand sizes; but the disadvantage is that the contact areas between the exoskeleton and the fingertip are quite limited and it is, thereby difficult to generate high force output. By full contact there are larger contact surfaces and closer interactions between the human hand and the exoskeleton, enabling more powerful assistance, enhanced working stability, and comfortable feeling during hand rehabilitation. Most existing hand exoskeletons have drawbacks in the clumsy control of the thumb. The human hand has a flexible thumb but most of the hand exoskeletons use only one actuator to control thumb movement (Li et al., 2016; Burns et al., 2019; Gasser et al., 2020). The flexion/extension of the fingers and the abduction/adduction of the thumb are two of the most important exercises to improve hand function (Gerez et al., 2020). An intriguing issue is how to design an exoskeleton with more than one actuator for the thumb to realize both flexion/extension and abduction/adduction.

Another issue is how to control the hand exoskeleton according to the movement intention of the patients. Surface electromyography (sEMG) is a non-invasive technology, which has been widely used in human-robot interaction and clinical examinations (Cote-Allard et al., 2019). The sEMG reflects muscle activations under the modulation of the central nervous system. Recording and processing of sEMG signals may help identify motion intention and provide key information for real-time control of hand exoskeleton (Li et al., 2018). Most exoskeleton hands controlled by sEMG adopt the strategy of mirror therapy principle, which suggests decoding the motor intention of the stroke patients from the non-paretic muscles because of their relatively normal function (Emerson et al., 2016). Several algorithms have been developed for sEMG processing, motion intention decoding, and exoskeleton control. But it is still challenging to realize online sEMG processing, time-varied motion intention, and real-time control for multiactuator exoskeletons.

This study presents a newly-designed wearable robotic hand exoskeleton with more active degrees of freedom (DOFs), larger range of motions (ROMs) for most joints, and the capability of being freely controlled by motion intention. The human finger is in full contact with the mechanical shells, and totally six linear actuators are adopted, generating high output forces for each digit. The thumb is controlled by two actuators to perform circumduction and adduction/abduction. The proximal interphalangeal (PIP) and distal interphalangeal (DIP) joints are driven by coupled links. Motion intentions for controlling the exoskeleton were decoded by the sEMG from the muscles of the non-paretic arm and hand. Patients could use this hand exoskeleton for repetitive training of grasping, pinching, individual finger control, thumb adduction/abduction, and thumb circumduction.

## Method

### Hand Exoskeleton Design

**Figure 1:**
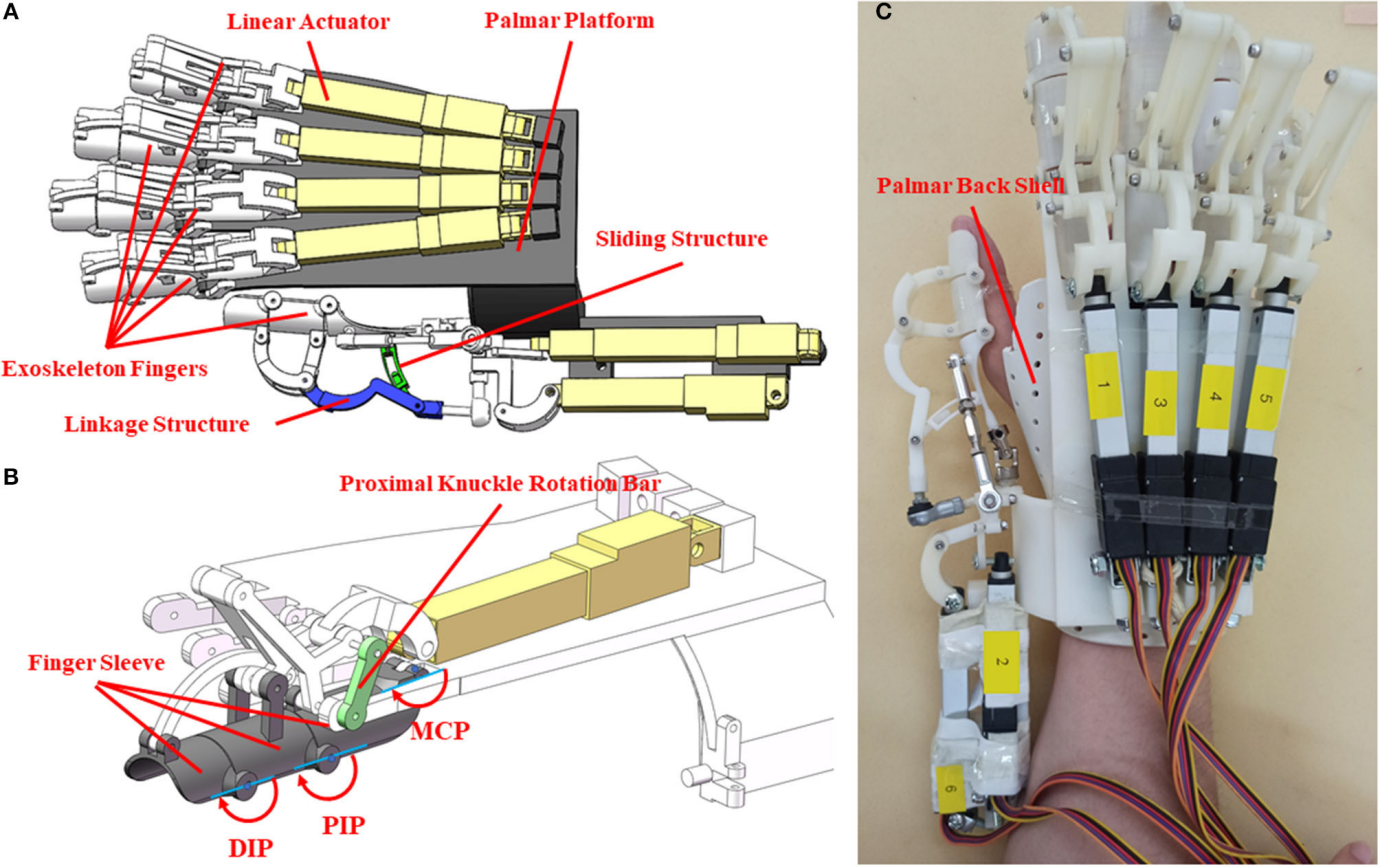
The mechanical design and realization of hand exoskeleton. **(A)** The mechanical design of the exoskeleton; **(B)** mechanical structure of exoskeleton index finger; **(C)** prototype of the hand exoskeleton.

The structure of the hand exoskeleton is shown in Figure 1. Thumb and fingers were connected to the palm back platform through a linkage. The finger was designed ergonomically following finger motion trajectories. The design allowed independent movement of the thumb and the four fingers, and circumduction, abduction, and the palm opposite for the thumb. Each finger was driven by a linear actuator, and the thumb was driven by two linear actuators. The mechanical structure was designed and simulated using Solidworks (Dassault Systems, USA), and was made from resin by 3D printing.

The palm back shell covering the wrist and the back of the hand was made of thermoplastic materials. The blank was first shaped and was then soaked in hot water to be softened up, fitting it to the hand of the users. The exoskeleton weighs 500 g, was fixed upon the palm back shell, and is convenient to wear. Six linear actuators (Actuonix, L12-50-210-12-p) with matching control boards were applied to drive the exoskeleton. The operating distance of the linear actuators was controlled by voltages. The linear actuator with a length of 102 mm and weight of 40 g can be bidirectionally driven.

**Figure 2:**
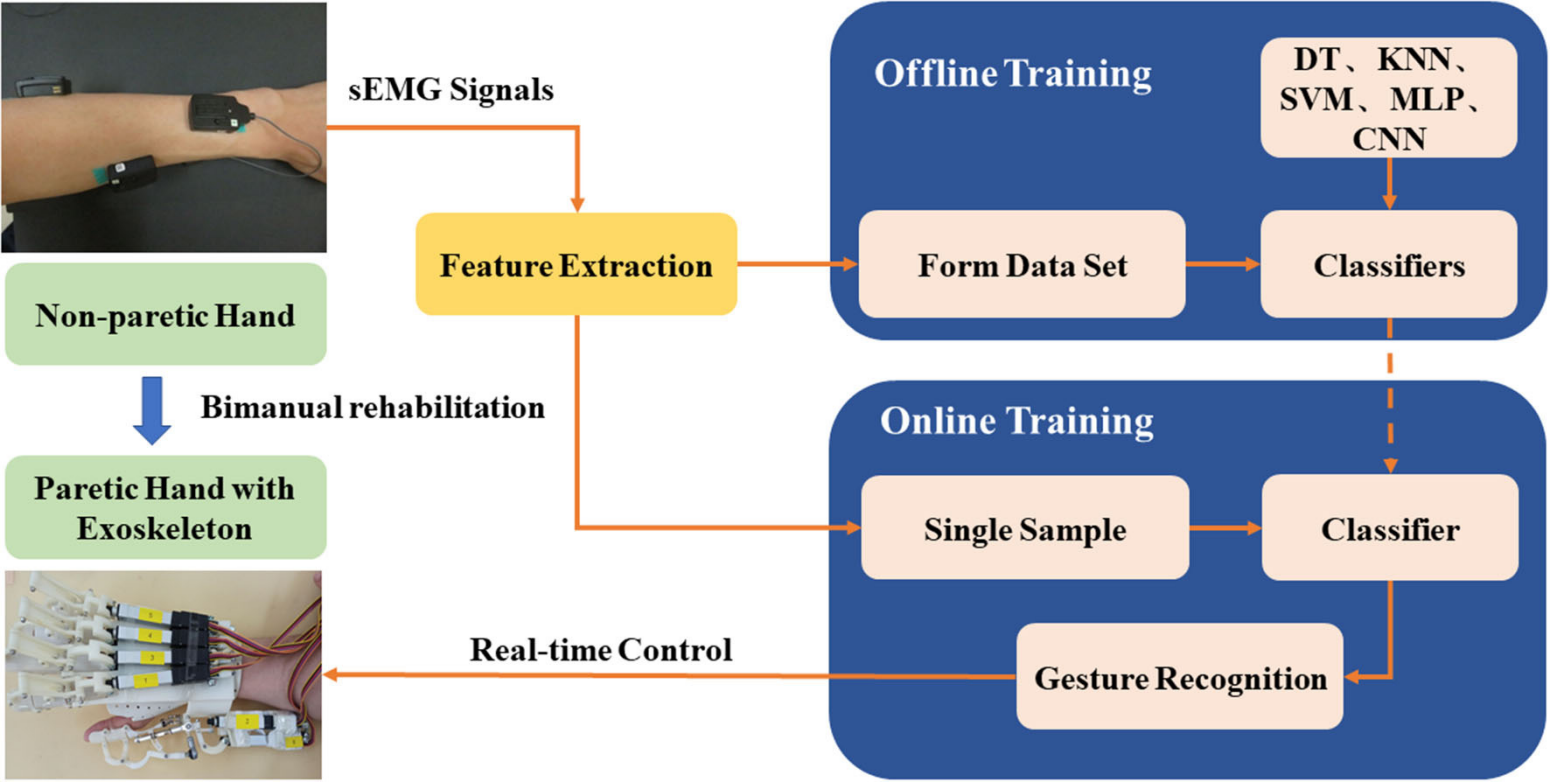
Schema of the control system for the hand exoskeleton based on non-paretic sEMG processing and offline-to-online classifications.

Each finger has three shells, connected by a linkage rod. As shown in Figure 1B, when the linear actuator reciprocates, the linkage rod transmits force to the finger shells, driving a motion for abduction/adduction. The exoskeleton fingers were designed following the anatomic characteristics of human fingers. Considering the thumb has higher DOFs than the fingers, the newly-designed exoskeleton adopted a more flexible structure for the thumb that can facilitate the thumb for inward rotation, grasping, and abduction. The force of the actuator can directly act on the carpometacarpal joint (CMC) or the metacarpophalangeal joint (MCP). When the force acts on the CMC joint, the wrist and palm joints move first, and as the CMC joint contacts the object, the movement of the CMC joint can be blocked so that the control of the tip of the thumb is not flexible enough. When the force acts on the MCP joint, the flexibility of the thumb could be increased, but this design is not suitable for grasping relatively bigger objects. The newly-designed exoskeleton did not follow any of these designs. Instead, the force of the new exoskeleton acts on the MCP and PIP joints of the thumb. The joint movement of CMC was driven by the friction of the sliding structure. The ROMs of the MCP and PIP joints were confined to avoid bending the grasped object. When the sliding structure moves, the CMC joint can be pushed forward until blocked by the grasping object. Then the force of the linear actuator accumulates on the MCP and PIP joints. The sliding part of the linkage structure helps extend the ROM of the thumb. Two linear actuators were used to control the movement of the thumb in the vertical planes so that the thumb can complete a circumduction. The schema of the control system for the exoskeleton is shown in Figure 2. This exoskeleton can be controlled by sEMG of the non-paretic forearm and hand following the mirror therapy principle. To examine the performance of the hand exoskeleton, the classifiers, and the real-time control, we set a sequence of experiments (specified in Experiment for hand exoskeleton performance to Experiment for real-time control of an exoskeleton). All these experiments were approved by the Institutional Review Board of Shandong University and were in accordance with the Declaration of Helsinki.

### Experiment for Hand Exoskeleton Performance

**Figure 3:**
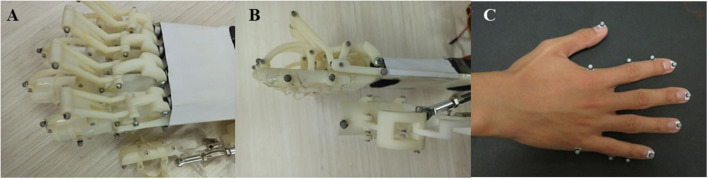
The reflective marker sets for kinematic analysis of the hand exoskeleton. **(A)** The top view of the marker sets; **(B)** the profile view of the marker sets; **(C)** the marker sets for the human hand.

The experiment was performed to examine the performance of the hand exoskeleton. A 3D motion capture system (Opti Track Motive, USA) with six cameras and clusters of reflective markers was used to collect the movement trajectories of the robotic finger joints. There were five reflective markers attached to the exoskeleton fingertips, six markers on the joints of the exoskeleton fingers, and three marks for coordinates of linear actuators (Figures 3A,B). Initially, all the digits of the exoskeleton were fully extended. Once a start command was received, the exoskeleton ran for a full movement cycle, that is, the thumb and the four fingers flexed to their extreme positions and then extended back to the initial positions. The test process consists of five cycles (Figures 3A,B). A representative subject participated in the experiment. The subject was instructed to perform flexion and extension of the thumb and fingers for five tries. The marker sets were demonstrated in Figure 3C. Specifically, five markers were attached to the fingertips, and six markers were attached to the joints of the index finger and little finger (Figure 3C). The movement trajectories of the fingertips and the index finger joint angles of the exoskeleton were calculated to evaluate the ROM of the exoskeleton. To calculate the joint angle, two adjacent markers formed a vector, and the joint angle was the angle between the two adjacent vectors. The ROMs were computed as the changes of joint angles from maximal flexion to full extension and compared with existing exoskeletons.

### Experiment for Classifier Selection

**Figure 4:**
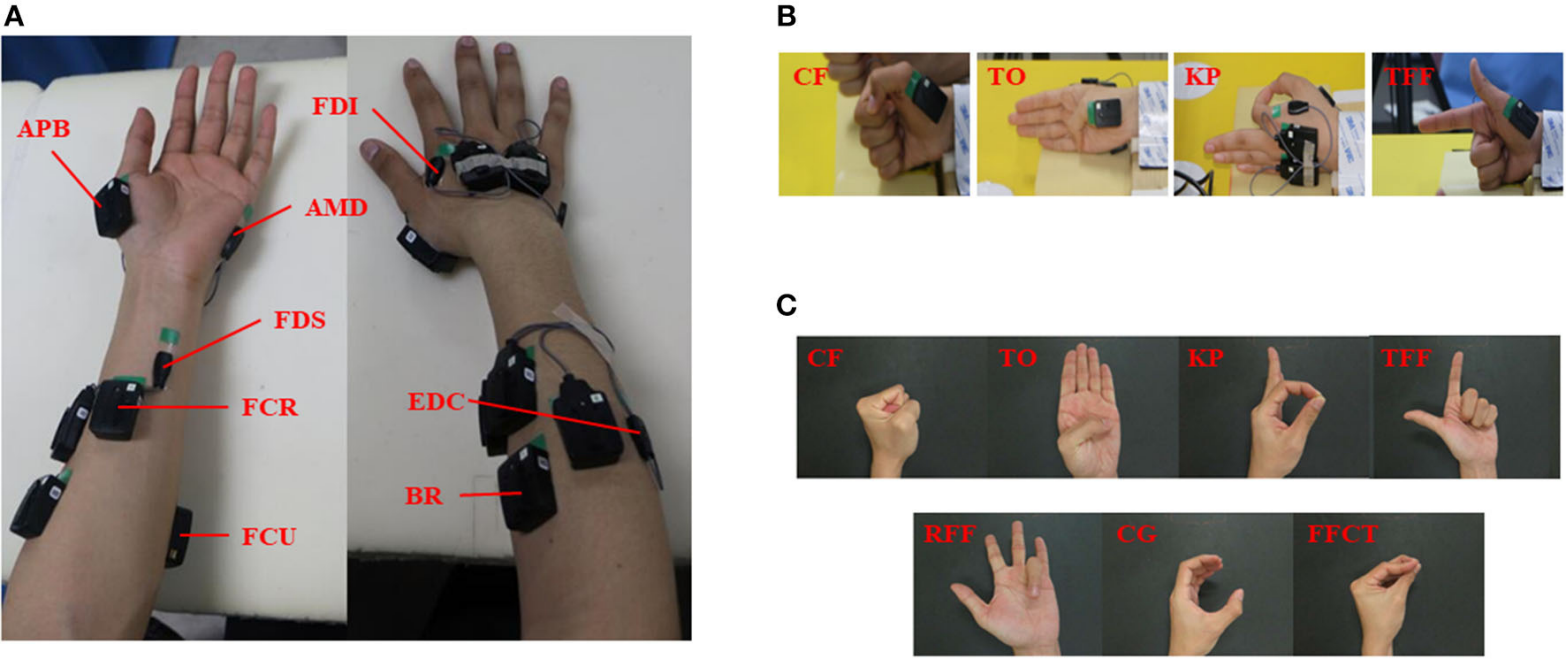
The muscles selection and hand gesture recognition. **(A)** The eight muscles of the bilateral forearms and hands; **(B)** the gestures for classifier selection; **(C)** the gestures for real-time control of exoskeleton.

Twenty-five healthy right-handed subjects (age = 23.2 ± 1.7 y, 12 women and 13 men) participated in the experiment. The subjects were informed of the purpose of the experiment and were given an informed consent form before the experiment. In total 16 muscles of the left and right hands were selected for the sEMG analysis. The muscles included the following: brachioradialis (BRA), flexor carpi ulnaris (FCU), flexor carpi radialis (FCR), extensor digitorum communis (EDC), flexor digitorum superficialis (FDS), abductor pollicis brevis (APB), first dorsal interosseous (FDI), and abductor digiti minimi (ADM), for both the left and right hands (Figure 4A). The sensors were attached to the muscle bellies, and the skin was cleaned with scrub and medical alcohol before attachment. The sEMG signals of the 16 muscles were recorded using the Trigno^TM^ wireless EMG system (Delsys, USA) at a sampling frequency of 1,000 Hz.

Four hand gestures, clenched fist (CF), thumb opposition (TO), key pinch (KP), and three fingers flexion (TFF) were selected as representative gestures in this experiment (Figure 4B). These gestures have been commonly used in hand gesture recognition in human-robot interaction and rehabilitation (He et al., 2017; Yu et al., 2018). The initial position for the four gestures was that the thumb should keep full extension whereas the fingers should be flexed. After hearing a command, subjects were instructed to perform a gesture with both hands and maintain this gesture for 8 s without much effort. The performances of the four gestures were randomized. Each gesture was repeated for 10 trials, with a rest period of 7–10 s between trials and 1–2 min between gestures.

To better understand the muscle activations of different gestures and provide a reference for muscle selection, a co-contraction index (CI) was calculated as follows:


(1) $$\begin{array}{r} CI=\frac{1}{T}\int_{T}A_{ij}\left(t\right)dt \end{array}$$

where, *A*_*ij*_ is the overlapping of sEMG envelopes of muscle *i* and muscle *j*, and *T* is the length of the sEMG envelope (Frost et al., 1997). The CI represents the level of the common contraction phase of the two muscles, ranging from 0 (no overlap) to 1 (full overlap) (Hu et al., 2009). The length of the sEMG segment was 500 ms, taken from the initial part of the datasets for each gesture. The sEMG signals subtracted the SDs of the envelopes of the averaged sEMG signals at the relaxed state and were then low-pass filtered using a fourth-order Butterworth filter with a cutoff frequency of 10 Hz. The data were standardized according to the maximum and minimum values of the data segments. The averaged CIs of the muscle pairs were calculated for each gesture, and then an 8 × 8 CI matrix was formed for the total eight muscles.

Five classifiers were used for hand gesture recognition. The sEMG signals were recorded from the BRA, FCU, FCR, EDC, FDS, APB, FDI, and ADM of the left forearm and hand. Five classifiers including support vector machine (SVM), K-near neighbor (KNN), decision tree (DT), multilayer perceptron (MLP), multichannel convolutional neural networks (multichannel CNN) were applied for classifying the CF, TO, KP, and TFF. A sliding window was used to extract features from the original signals. The window width was 128 ms and the sliding distance was 78 ms. We selected 13 specific features including zero-crossings (ZC), root mean square (RMS), mean absolute value (MAV), waveform length (WL), variance (VAR), slope sign change (SSC), Willison amplitude (WAMP), mean value (MEAN), the standard value (STD), mean frequency (MNF), median frequency (MF), mean power frequency (MPF), and Lempel-Ziv complexity (LZC). The ZC, RMS, MAV, WL, VAR, SSC, and WAMP have commonly used time-domain features (Hua et al., 2020; Qu et al., 2020; Wu et al., 2020; Duan et al., 2021). In addition to these parameters, we further calculated the MEAN and STD, so that the time-domain features reached nine. Considering the limitation that the time-domain parameters are vulnerable to the noise or interference to amplitudes, the frequency-domain features were selected, including MNF, MF, and MPF. Furthermore, LZC was selected to examine the non-linear characteristics of the signals. All these parameters could provide abundant information with low computing costs and good real-time performance. Among all the sEMG data, we used 80% of the data as the training set and the other 20% for verification. The classifiers were trained based on the sEMG signals for each subject individually.

The SVM with a linear kernel that could easily deal with high dimensional representation was used (De Smedt et al., 2019). The details of SVM are as follows: a one-vs-rest strategy was used and a G-binary-classifier was obtained, where G was the number of different gestures in the experiment. The KNN is a machine learning classification algorithm, which calculates the distance to all the training samples, and selects the k-closest samples (Amin et al., 2019). The DT builds classification or regression models in the form of a tree structure. It breaks down a dataset into smaller and smaller subsets while at the same time an associated DT is incrementally developed. According to the DT algorithm, the final result is a tree with a great many decision nodes (Shengchang et al., 2017). In this study, the split criterion was the Gini index and the maximum number of splits was 100.

**Figure 5:**
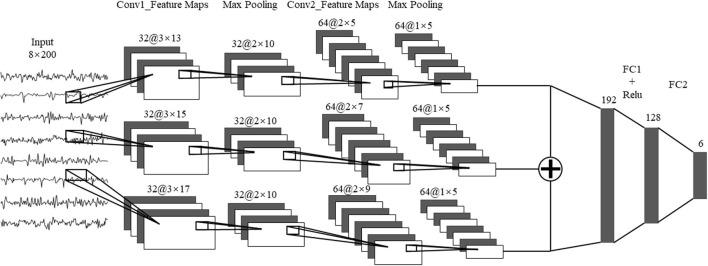
The multi-channel CNN algorithm. Conv1, convolution layer 1; Conv2, convolution Layer 2; FC1, fully connected layer 1; FC2, fully connected layer 2.

The network of MLP included an input layer with 104 nodes, two hidden layers constructed by 40 nodes for each layer, and an output layer with six nodes. The MLP followed a back propagation (BP) algorithm. The infrastructure of CNN included a convolution layer and a fully connected layer. The sEMG signals were first transformed into a series of images for the CNN network. For the eight muscles, each sEMG was segmented with 200-ms windows with a sliding distance of 150 ms, thereby generating 200 × 8 images. The images were input into the convolutional layer to extract features and, thus, reduce the dimension followed by a classification from the fully connected layer. The CNN network could be expanded to multiple channels, which could extract more patterns out of the sEMG signals. The structure of multichannel CNN was shown in Figure 5. Each channel has two convolutional layers and two pooling layers, with different convolution kernels. A max-pooling was used for the CNN to reduce the dimension of the convolutional features. A rectified linear unit (ReLU) was used as an activation function for the fully connected layer. The parameter of the dropout layer was 0.5 to avoid over-fitting by randomly deleting the redundant parts of the hidden layer.

### Experiment for Real-Time Control of the Exoskeleton

Five healthy subjects (age: 23.4 ± 1.9 year, one woman and four men) participated in the experiment. Six wireless sEMG sensors were attached to six muscles of their left hand, including the EDC, FCU, FCR, BRA, FDS, and FDI. Subjects sat in front of a testing table with their left hand laid on the table in a relaxed state. In total seven hand gestures (as shown in Figure 4C) were tested, including CF, TO, KP, and TFF. Four gestures were performed in the prior offline tests, and three new gestures which support grasping and manipulation and are commonly used in hand rehabilitation training were added: ring finger flexion (RFF), cylindrical grip (CG) and fingertips closed together (FFCT) (Zheng et al., 2011; Chen et al., 2017; He et al., 2017; Yu et al., 2018). The performance of each gesture included three phases: a 3-s relax, a 77-s action phase, and a 3-s relax phase. When performing the hand gestures, the subject was not allowed to produce high-level force to maintain the gesture. Subjects were given enough time for rest between trials and sessions. Each gesture was repeated for three trials. After the classifier was trained through offline classification, subjects were instructed to perform two trials for the real-time classification.

The raw sEMG signals were recorded simultaneously at a sampling frequency of 1,000 Hz. The sEMG signals were analyzed using the siding window technique that the window size was 128 ms without overlap at the adjacent windows. About 54 classification results were achieved in each trial. The same 13 features as the abovementioned offline classification were selected. The SVM was used for classification. To distinguish the relaxation state from the gesture execution state, a threshold was set for the relaxation state using the absolute value of Teager Kaiser energy (TKE) (Solnik et al., 2010). The formula for calculating the absolute values after TKE treatment is as follows:


(2) $$\begin{array}{r} x_{n}=|x_{n}^{2}-x_{n-1}x_{n+1}| \end{array}$$

where *x*_*n*_ is the sample point of sEMG signals, *x*_*n*−1_ and *x*_*n*+1_, are the former and the latter sampling points, respectively. To increase the classification performance, the classification results were verified three consecutive times. If the results were the same all three times, then the classification results could be accepted. Otherwise, the classification analysis was performed again. The programs for sEMG processing, classifiers, and real-time control were realized using MATLAB (MathWorks, USA).

## Results

**Figure 6:**
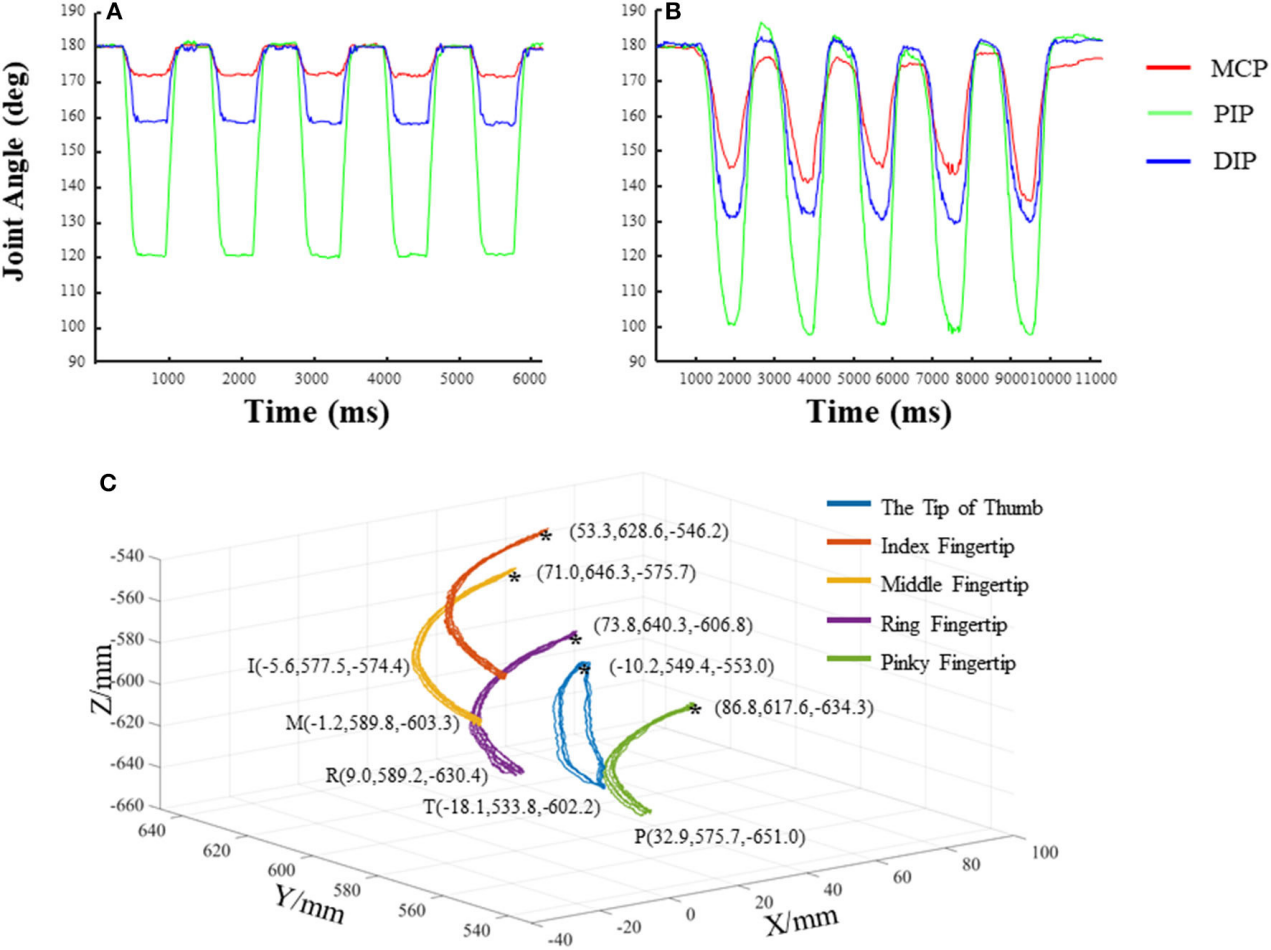
The joint angles and trajectories of the exoskeleton and human digits during flexion and extension. **(A)** The joint angles of the metacarpophalangeal joint (MCP), PIP, and DIP of the exoskeleton index finger; **(B)** the joint angles of the MCP, PIP, and DIP of the index finger of a representative subject; **(C)** the trajectories of the exoskeleton fingertips.

Figures 6A,B demonstrate the joint angles of the MCP, PIP, and DIP of the exoskeleton and the index finger of a human subject during flexion and extension, respectively. Results showed that the MCP, PIP, and DIP joints of the exoskeleton index finger at maximal flexion were 171.26°, 119.57°, and 157.56°, respectively. The ROMs of the MCP, PIP, and DIP joints of the exoskeleton index finger were 8.74°, 60.43°, and 22.44°, respectively. Variations of the joint angles were small, showing good repeatability. The ROMs of the exoskeleton of the current study were compared with those of previous studies (Table 1) (Iqbal et al., 2014; Kim et al., 2017; Refour et al., 2019). The PIP and DIP joints of the new exoskeleton showed larger ROMs, but its MCP joint showed smaller ROM than those of the previously designed exoskeletons. The new exoskeleton set two activate DOFs for the thumb, with one more DOF for the thumb than the previously designed exoskeleton. This new design enables thumb circumduction, in addition to the adduction/abduction for the thumb, compared with the previous exoskeleton. Figure 6C shows the movement trajectory of fingertips during digit flexion and extension of the hand exoskeleton. The trajectories of the five testing cycles overlapped, demonstrating a relatively stable motion performance and good repeatability from trial to trial. The trajectory of the thumb presents that the exoskeleton could assist the thumb for circumduction (Figure 6C).

**Table 1:** Comparison between the new exoskeleton and some previous results.

**Exoskeletons**	**Finger Number**	**Total Activate DOFs**	**Activate DOFs of Thumb**	**ROM of Index Finger**		
				**MCP**	**PIP**	**DIP**
This study	5	6	2	8.74°	60.43°	22.44°
Iqbal et al. (2014)	2	2	1	<30°	NA	NA
Refour et al. (2019)	5	6	1	35°	21°	13°
Kim et al. (2017)	5	5	1	≈13°	≈21°	<5°

**Figure 7:**
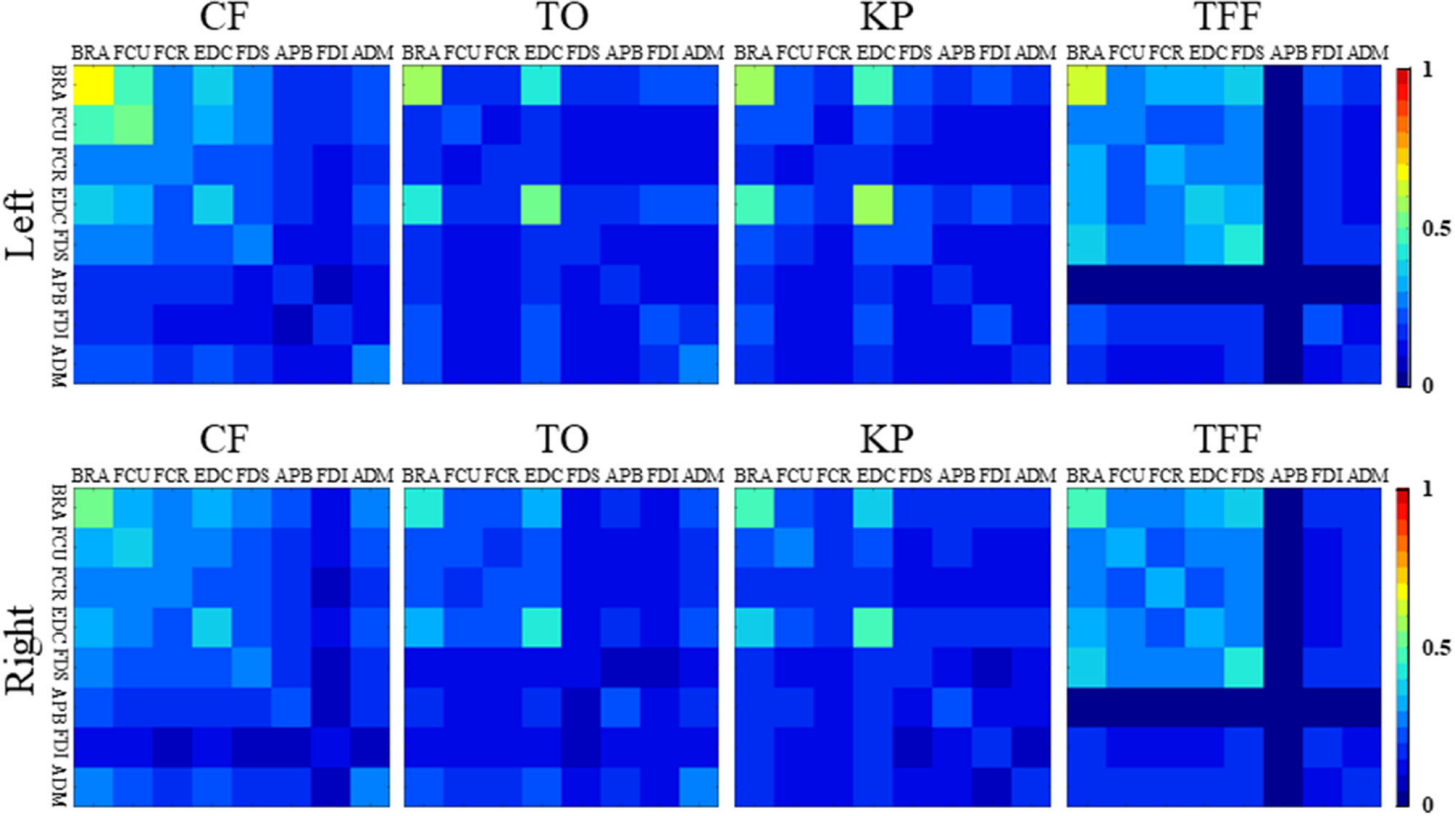
The co-contraction index (CI) matrices for the four gestures of the left and right hands.

Results of sEMG analyses in Figure 7 showed the following: (a) the averaged coordination matrices of the same hand gesture were similar; (b) the coordination matrices among the forearm muscles showed constant values across the different target gestures than those among the hand muscles; and (c) the matrices among the BRA, FCU, EDC, and FDS were different from those among the muscles of the APB, FDI, and ADM. The CI values of the forearm muscles were higher while performing the CF and TFF gestures compared with the other gestures. The CI values between the APB and the other muscles were lower for the TFF, suggesting that lower intermuscular coordination than the other muscle pairs for this gesture. The FDS had similar results as performing the TO gesture. The BRA and EDC showed greater values for CF, TO, KP, and TFF. There were high correlation coefficients (which were 0.935, 0.903, 0.928, and 0.978 for CF, TO, KP, and TFF) between the coordination matrices of the left and right hands for the same hand gestures, indicating that the muscles of both hands were activated following similar patterns.

**Figure 8:**
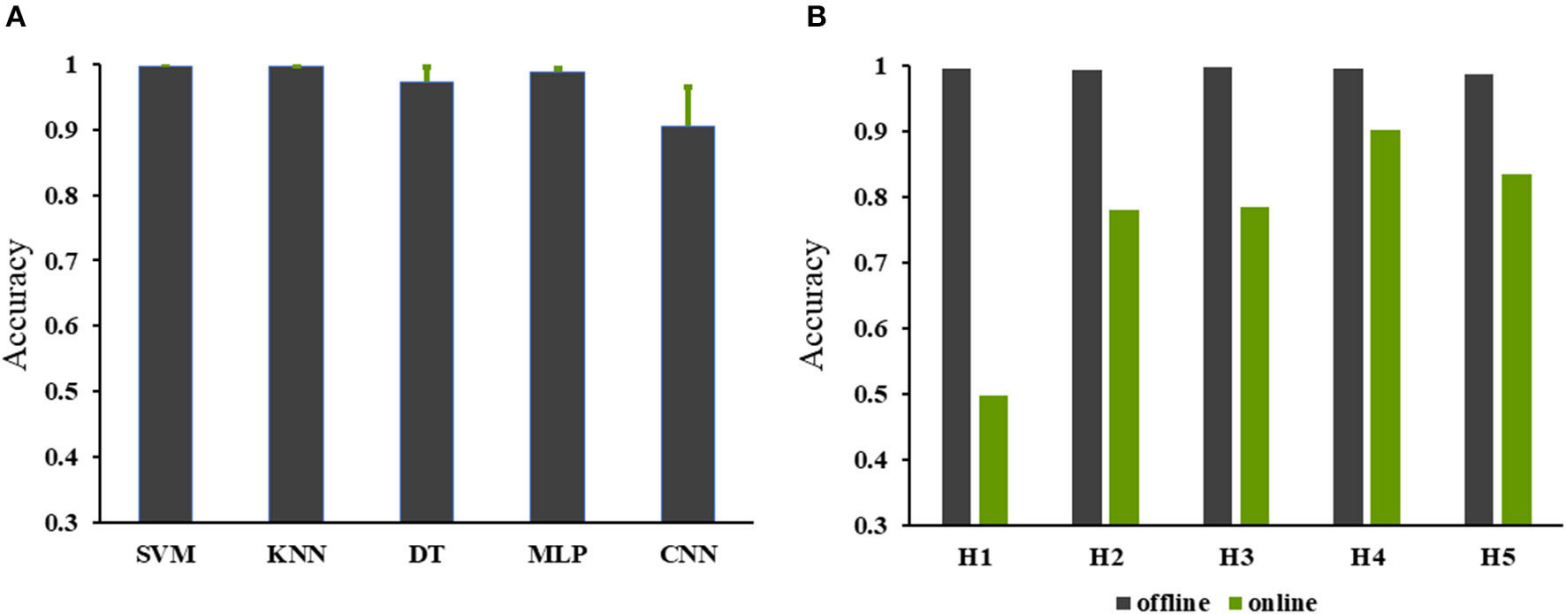
The online and offline classification accuracies. **(A)** The accuracies of the offline classification for classifier selection; **(B)** the accuracies of the online and offline classifications for real-time control of exoskeleton using the support vector machine (SVM).

**Figure 9:**
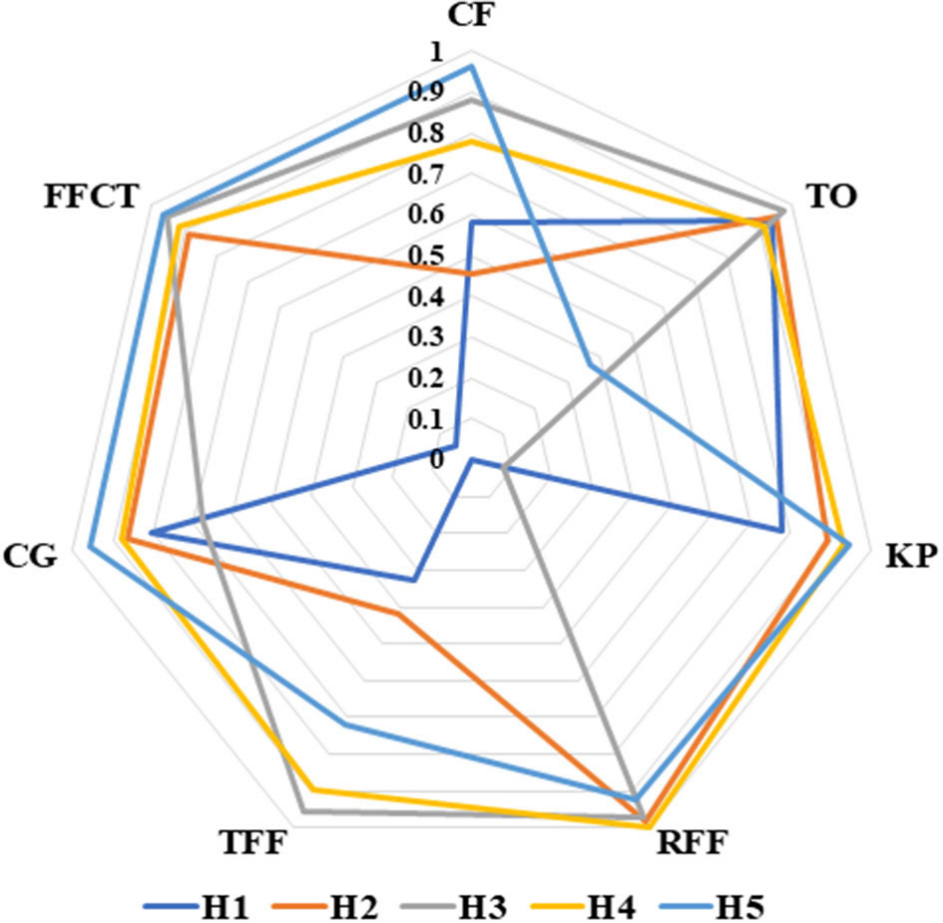
The subject-specific online classification accuracies for recognizing the hand gestures.

**Figure 10:**
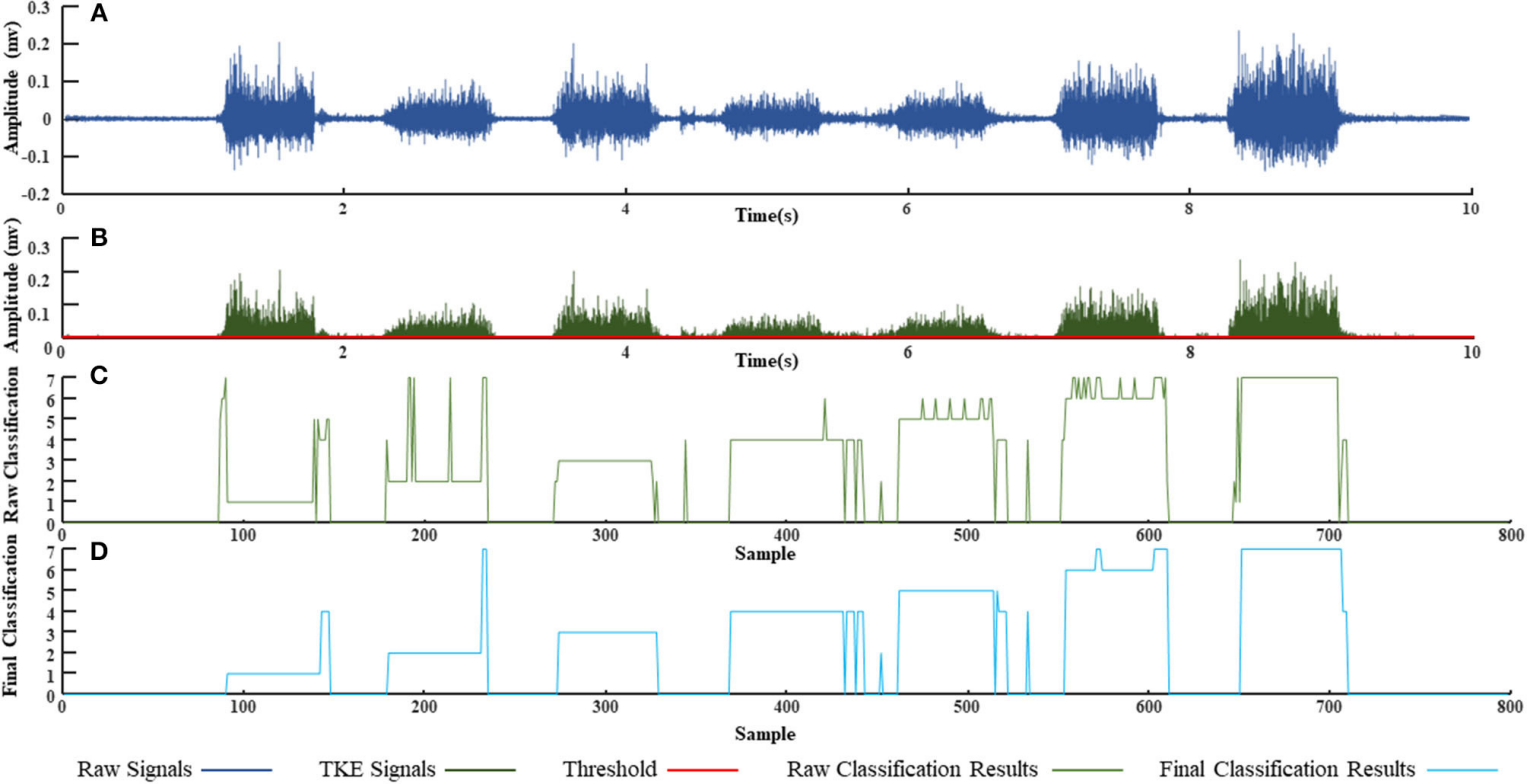
Real-time hand gesture recognition from a representative subject (H4). **(A)** The raw surface electromyography (sEMG) signals recorded from the left brachioradialis (BRA); **(B)** the Teager Kaiser energy (TKE) signals extracted from **(A)** with a threshold 0.001 mv; **(C)** the raw classification based on the SVM; **(D)** final classification based on the TKE and the three consecutive judgment algorithms.

Results of offline classification accuracies for the 25 subjects are shown in Figure 8A. The SVM and KNN showed classification accuracies up to 99%; the MLP and DT showed classification accuracy over 95%; whereas the CNN showed accuracy just over 80%. Results of real-time control are demonstrated in Figure 8B. The offline classification achieved much higher accuracies than the online classification among the five subjects. The online classification accuracy of a subject (H4 subject in Figure 8B) was higher than 90%. The online classification accuracies for the H2, H3, and H5 subjects were about 80%, but the accuracy for the H1 subject was lower than 50%. Figure 9 shows the classification accuracies of the seven hand gestures for the five subjects. In general, the classification accuracies for H3, H4, and H5 were better than the other subjects, and the TO and CG gestures achieved better classification performance. Recognitions for FFCT, RFF, and KP gestures of the four subjects were higher than the other gestures except for H1. Figure 10 demonstrates the original sEMG signals and the TKE signals of the BRA from a representative subject (H4). The final classification based on TKE signals and the three consecutive judgment algorithms had better performance than the classification based on the raw sEMG signals. The threshold was 0.005 mv. By removing the peaks out of the original classification results, although the calculation time for gesture classification increased about 300 ms, the final classification results showed better reliability than the raw classification. The real-time control of the exoskeleton used motion intention extracted from sEMG is shown in a video; URL: https://figshare.com/s/b3a2a1f3ac43172aba76.

## Discussion

In this study, we developed a novel wearable robotic hand exoskeleton with multijoints, more active DOFs, larger ROMs for most joints, and the capability of being freely controlled by the motion intention. This hand exoskeleton is capable of driving the thumb and four fingers independently and meets the needs of hand function rehabilitation. Two linear actuators drive the exoskeleton thumb, facilitating a more natural movement of the thumb of the patients for abduction/adduction and circumduction. In addition, the mechanical structure of the exoskeleton could realize the hand functions such as grip and pinch. Finger circumduction is the most difficult movement in exoskeleton design because it requires the coordination of two actuators in different directions. The changes of the exoskeleton index finger angles in five cycles were similar to those of a human hand. The similarity of the movement of the five cycles was very high, indicating that the exoskeleton movement could be reliable for motion training.

The exoskeleton in this study showed higher ROMs of the PIP and DIP joints compared with the other exoskeletons, except for the MCP joint that showed a lower ROM than the previously designed exoskeletons. This design was inspired by the human hand anatomic and functional characteristics. Traditionally, exoskeletons mainly exert force on the MCP joint, resulting in relatively larger ROM for the MCP but smaller ROM for the PIP and DIP. During human grasping, the ROM of the PIP and DIP joints are relatively higher than those of the MCP. We, thus, increased the ROMs of the PIP and DIP joints but restricted that of the MCP joint. Although the ROM of the MCP joint of the new exoskeleton was smaller than the other exoskeletons, changes of joint angles during flexion and extension were similar to the human hand.

Results of hand exoskeleton control based on the sEMG signals showed that the classification accuracies were high. Specifically, the H2 subject showed high classification accuracies for the FFCT, CG, RFF, KP, and TO; the H3 subject had high accuracies for the CF,FFCT,TFF,RFF, and TO; the H4 subject had high accuracy for the FFCT, TFF, RFF, KP, and TO; and the H5 subject showed high accuracies for the CFF, FFCT, CG, RFF, and KP. However, for each subject, there were one or two actions that could not reach a high accuracy, such as the CF and TFF for H2, KP for H3, TO for H5, which decreased the overall classification accuracies. The accuracy of online classification for H1 was not ideal, but consistent with the previous studies where there were still three actions (TO, CG, KP) that had high accuracies (Furui et al., 2019). The classification accuracies for each subject were not the same, and thus, different classifiers should be selected and applied individually (Xiloyannis et al., 2017; Dwivedi et al., 2019). For the same subject, the accuracies for classifying different actions could also be quite different. There were between-subject differences in muscle contractions, suggesting that each individual may perform the same action by activating the muscles in quite a different way. The accuracy of predicting the same hand gesture could be different for different individuals (Cote-Allard et al., 2019; Parajuli et al., 2019).

According to the results of real-time control, the online accuracy was not as high as the offline classification, which is consistent with the previous studies showing that the online accuracy was a more challenging issue of the sEMG controlled hand exoskeleton (Chen et al., 2019; Parajuli et al., 2019). Previous studies showed that in the process of stable grasping, compared with the muscles innervated by different nerves, the muscles innervated by the same nerve showed lower sEMG signal coherence (Pasluosta et al., 2013). For the muscles selected in this study, the BRA and EDC were innervated by the radial nerve, and the FCU and FDI were innervated by the ulnar nerve. Thus, the FCR, FDS, APB, and ADM were innervated by the median nerve. Different from the previous studies, the current study computed the first 500 ms datasets in the execution process instead of the stable grasping data. For all gestures, muscles innervated by the same nerve showed lower CI values compared with the muscles innervated by different nerves (Figure 7).

Considering most hand usage in daily activities was under visual supervision, visual feedback was not removed from the experiment. Also, because all the subjects equally received visual feedback during hand performance, the potential effects of visual feedback on results could be further limited. The objective of the current study was to demonstrate a newly-designed wearable robotic hand exoskeleton with more active DOFs, larger ROMs for most joints, and the capability of being freely controlled by motion intention. However, because this is a preliminary study showing a novel design of an exoskeleton, more work is needed prior to any clinical tests. We aim to perform a clinical study in the near future to show the performance of this new exoskeleton for patients with neuromuscular disorders, such as in stroke patients.

## Conclusion

In this study, we developed a new wearable robotic hand exoskeleton with multiple joints, more DOFs for the thumb, and larger ROM. We also investigated the control of the hand exoskeleton based on the sEMG signals. The former provides a platform and the later builds up its control system. Considering that the post-stroke patients have difficulty in controlling their paretic hands, we adopted the strategy of mirror therapy principle, by which the motion intention was decoded based on the sEMG signals of the non-paretic upper limb and hand. We applied machine learning and deep learning methods to verify the sEMG offline classification. The exoskeleton engaged six linear actuators, in which two were for the thumb and four for the fingers, and can realize independent movement by each digit and the coordinative movement by multiple fingers for grasp and pinch. The joint angles of the exoskeleton index finger were comparable to those of the human index finger, and the circumduction of the thumb was maintained stably. For the real-time control, three out of the five subjects showed an accuracy of about 80%, and one subject showed an accuracy over 90%. The control strategy based on sEMG classification has been integrated with the newly-designed exoskeleton system. This new wearable exoskeleton may play a role in hand rehabilitation in post-stroke patients and may advance the dexterous exoskeleton control according to the motion intention of the patients.

## Data Availability Statement

The datasets presented in this study can be found in online repositories. The names of the repository/repositories and accession number(s) can be found below: URL: https://figshare.com/s/b3a2a1f3ac43172aba76.

## Ethics Statement

The studies involving human participants were reviewed and approved by Shandong University Qilu Hospital Ethics Committee. The patients/participants provided their written informed consent to participate in this study.

## Author Contributions

KL: original motivation and idea, resources and financial support, design and realization, and writing and revision of the manuscript. ZL: writing of the manuscript, control of hand exoskeleton, signal processing, and classification. HZ: design of the hand exoskeleton, experiment and data analysis, and writing of the manuscript. NW: resources, financial support, and revision of the manuscript. All the authors contributed to the article and approved the submission.

## Funding

This study was supported by the National Natural Science Foundation of China (62073195), the National Key Research and Development Program (2020YFC2007904), and the Key Research & Development Programs of Guangdong Province (2020B0909020004) and Shandong Province (2019GSF108164, 2019GSF108127 and 2019JZZY021010).

## Conflict of Interest

The authors declare that the research was conducted in the absence of any commercial or financial relationships that could be construed as a potential conflict of interest.

## Publisher's Note

All claims expressed in this article are solely those of the authors and do not necessarily represent those of their affiliated organizations, or those of the publisher, the editors and the reviewers. Any product that may be evaluated in this article, or claim that may be made by its manufacturer, is not guaranteed or endorsed by the publisher.
